# Dietary macrominerals: Updated review of their role and orchestration in human nutrition throughout the life cycle with sex differences

**DOI:** 10.1016/j.crfs.2023.100450

**Published:** 2023-02-01

**Authors:** Mohamed A. Farag, Bishoy Abib, Zhiwei Qin, Xiaolei Ze, Sara E. Ali

**Affiliations:** a Pharmacognosy Department, College of Pharmacy, Cairo University, Cairo, Egypt, Kasr El Aini St, P.B, 11562, Egypt; b Chemistry Department, School of Sciences & Engineering, The American University in Cairo, New Cairo, 11835, Egypt; c Center for Biological Science and Technology, Advanced Institute of Natural Sciences, Beijing Normal University at Zhuhai, Zhuhai, Guangdong, 519087, China; d Macau University of Science and Technology Zhuhai MUST Science and Technology Research Institute, Zhuhai, Guangdong, China; e BYHEALTH Institute of Nutrition & Health, No.3 Kehui 3rd Street, No.99 Kexue Avenue Central, Huangpu District, Guangzhou, Guangdong, 510663, China; f Department of Pharmaceutical Biology, Faculty of Pharmacy & Biotechnology, The German University in Cairo, Egypt

**Keywords:** Minerals, Macronutrients, Calcium, Phosphorus, Magnesium, Sulfur, Sodium, Potassium, Life cycle, Sex, Deficiency

## Abstract

Macrominerals play vital roles in a multitude of physiologic systems. A myriad of biochemical reactions are dependent on or affected by these electrolytes. The current review attempts to identify the role of macrominerals as calcium, phosphorus, magnesium, sodium, potassium and sulfur in human health, in addition to their absorption and homeostasis inside the body. We also focused on their amount in major food sources and the recommended daily intake of each macromineral. In addition, a deep insight into the orchestration of the 6 different macrominerals’ requirements is presented across the human life cycle, beginning from fertility and pregnancy, and reaching adulthood and senility, with insight on interactions among them and underlying action mechanisms. The effect of sex is also presented for each mineral at each life stage to highlight the different daily requirements and/ or effects. The current review identified the role of macrominerals in human health, in addition to their absorption and homeostasis in the body. Based on the in-depth understanding of the factors influencing the metabolism of macrominerals, we could better explore their safety and possible therapeutic potential in specific disorders. There is still a need to precisely demonstrate the bioavailability of macrominerals from various types of functional food.

## Highlights

• Macrominerals s are essential for numerous body homeostatic functions.
• The orchestration of Macrominerals requirement across the human life is presented.
• Insight on the interactions among minerals and their action mechanisms.
• Effect of sex is also discussed for each micronutrient for each life stage.

## Graphical abstract

**Figure:**
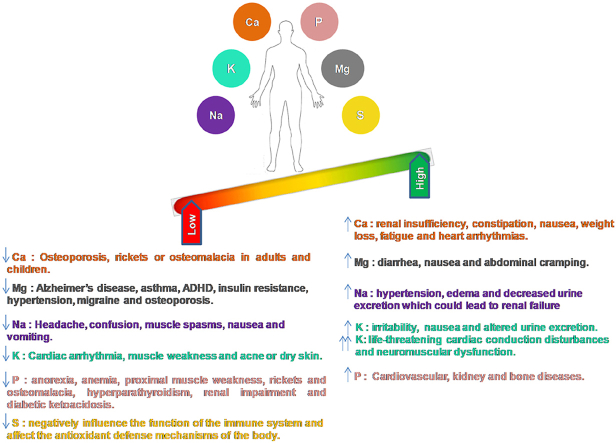

## 1 Introduction

The human body needs about twenty different minerals in order to function properly. These minerals are usually classified into two main categories that are the micro- and macro-minerals depending on their required daily intake rather than their relative importance or physiological functions (Gupta and Gupta, 2014; Morris and Mohiuddin, 2021). Macrominerals are typically needed at levels higher than 100 mg/day to include calcium (Ca), phosphorus (P), magnesium (Mg), sulfur (S), sodium (Na) and potassium (K). On the other hand, microminerals are needed in amounts lower than 100 mg/day and to include elements such as iron (Fe), zinc (Zn), iodine (I), selenium (Se), manganese (Mn), chromium (Cr), copper (Cu), molybdenum (Mo), fluorine (F), boron (B), cobalt (Co), silicon (Si), aluminum (Al), arsenic (Ar), tin (Sn), lithium (Li) and nickel (Ni) (Morris and Mohiuddin, 2021). Macrominerals are required by the body to sustain its basic functions and are optimally obtained by eating a balanced diet. In fact, they are essential for a wide variety of metabolic and physiological processes in the human body, especially in homeostasis as well as metabolism, along with their well-known importance for proper functioning organs and overall good development and growth of our bodies. Many medical conditions could arise in cases of excessive intake or deficiency (Morris and Mohiuddin, 2021). This review is an attempt to illustrate and provide an in-depth discussion on the role of macrominerals in our bodies. Their health benefits, levels in selected food sources, exact required daily intake of each macromineral will be covered, as well as, the risks of having more or less intake. We further highlighted the significance of macrominerals throughout the different human life cycle and in context to different sex type for each mineral as outlined in the next subsections.

## 2 Methods

Clinicians and nutritionists mainly benefit from review articles to update their knowledge in their field of specialization, use these articles as an up-to-date guideline and employ the best scientific information available to apply to clinical practice (Gülpınar and Güçlü, 2013). Herein, we aimed at conducting a literature review which is conceptualized as the ‘gold standard’ since it provides a detailed and comprehensive literature surveying on an emerging topic. An extensive search was performed on Web of Science, PubMed and Scopus electronic databases using database-specific search terms in two broad areas “macrominerals, macroelements, calcium, phosphorus, magnesium, sodium, potassium and sulfur + fertility, childhood/children, pregnancy, adults, elderly” and “calcium, phosphorus, magnesium, sodium, potassium and sulfur + required daily allowance”. The inclusion criteria mainly focused on the role of sex differences and age on the recommended daily allowance of macrominerals. In addition, the health benefits and risks associated with the decrease or increase in macrominerals' level throughout the human life cycle and different sex. Data extraction was performed, followed by compilation and comparison of all recommendations to identify the key consistent recommendations. Owing to discrepant findings across primary studies and the significant high number of articles in this field, each citation was independently screened using title and abstract. Only the relevant citations, review articles and original researches then underwent a second stage of full text screening. Exclusion criteria included papers preprints, conference proceedings and papers for which only abstract was found. Finally, the reference lists of retrieved key papers were also examined for articles of relevance. It should be noted that animal studies were only included in case there were no or few human studies. The number of identified articles by searching the keywords of each “mineral" and “life cycle" or “sex" in title were 220, 41, 40, 13, 148 and 64 for calcium; phosphorus; magnesium; sulfur; sodium; potassium, respectively. Following the exclusion criteria described above, the number of compiled papers reached 140 cited in text. In this review, we aimed at looking on the orchestration of the main elements requirement across the human life cycle beginning from fertility and pregnancy, passing through to the infancy, childhood, adolescence, and reaching to adulthood and senility, with insight on the possible interactions among them. Emphasis is also given towards the role of different minerals throughout the life cycle, symptoms associated with deficient or excessive levels and typical management for each element, with future perspectives. The effect of sex differences is further discussed for each micronutrient at different life stages to highlight for the different daily requirements.

## 3 Results and discussion

### 3.1 Sodium and potassium

#### 3.1.1 Sodium and potassium functions, sources, dietary intake and bioavailability

**Fig. 1:**
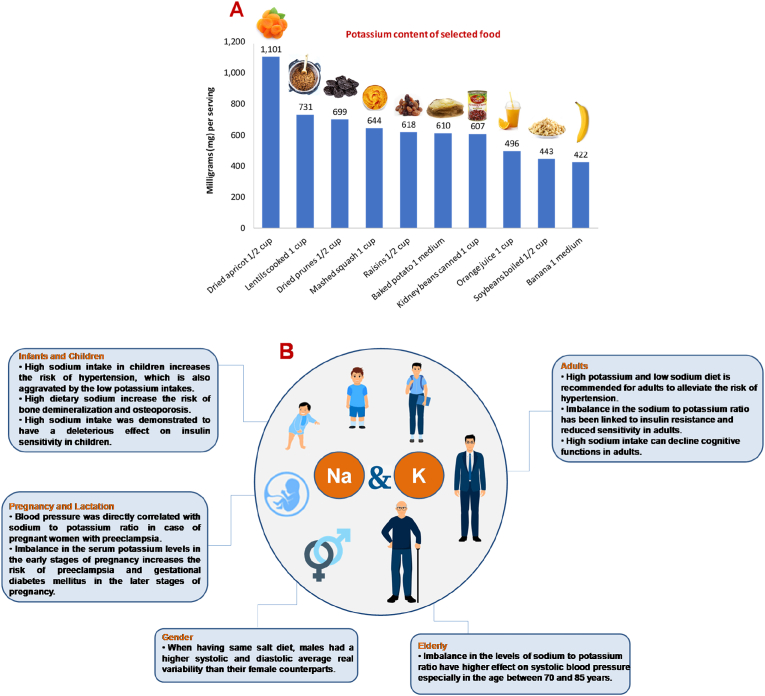
(A) Potassium content of selected food (B) Significance of sodium and potassium throughout the human life cycle and sex effect.

Sodium and potassium are two essential macrominerals required by the body to maintain cellular homeostasis, metabolism and many other functions. Most metabolic processes are dependent on or affected by these electrolytes. Among the functions of these electrolytes is maintenance of normal pH in extracellular fluid, regulate osmotic pressure and water distribution in various body fluid compartments, act as cofactors for many enzymes and are involved in oxidation-reduction reactions (Pohl et al., 2013). Potassium is necessary for the proper function of all cells, tissues and organs in the human body. In fact, it is essential for heart function and plays a key role in skeletal and smooth muscle contraction, thus it is essential for normal digestive and muscular function. To maintain proper electrolyte balance, water, sodium, and potassium are in constant exchange between the intracellular and extracellular fluid compartments (Pohl et al., 2013; Medicine, 2006; Reynolds et al., 2006). Compared to sodium, potassium usually enters the cell more readily and initiates the sodium-potassium exchange across the cell membranes. In the nerve cells, this sodium-potassium flux generates the potential gradient required for the conduction of nerve impulses which in turn initiates muscle contraction and regulates the heartbeat (Gupta and Gupta, 2014; Pohl et al., 2013; Reynolds et al., 2006). Sodium on the other hand is considered as the major cation in blood and extracellular fluids with a vital role in the absorption of other macronutrients as amino acids, water and sugars (Medicine, 2006; Goff, 2018). Dietary requirements for sodium and potassium vary widely, but generally, daily intake should be in small amounts. Sodium intake averages at ca. 4 g/day in developed countries, which is twice the maximum daily intake of 2 g/day as recommended by the World Health Organization (WHO) (Thout et al., 2019). On the contrary, potassium is often consumed in suboptimal levels. The WHO recommends a potassium intake of ca. 3.5 g/day. However, in chronic kidney disease populations, intake is estimated to average around 2.4 g/day (Leonberg-Yoo et al., 2017). Data showed that maintaining sodium to potassium ratios can have a positive impact on health and reduces the risk of many diseases i.e., hypertension, renal and cardiovascular disease. In fact, studies showed a strong link between these two macrominerals, where higher levels of sodium and lower levels of potassium intake are associated with higher blood pressure. However, the shape and magnitude of these associations can vary by studying participant characteristics or intake assessment method. Therefore, patients at risk of cardiovascular and renal disease are urged to adhere to the recommended daily intake. Nevertheless, this ratio is hard to maintain given that modern dietary is characterized by high sodium and low potassium intake (Weaver, 2013; Stamler et al., 1989). Adequate monitoring of intake is essential to guide dietary advice in clinical practice and can be used to investigate the relationship between intake and health outcomes. The adequate intakes (AI) for sodium and potassium are outlined in Table 1 (Medicine, 2006; Oria et al., 2019). The AI is a recommended average daily nutrient intake level, based on experimentally derived intake levels or approximations of observed mean nutrient intake by a group (or groups) of apparently healthy people that are assumed to be adequate. AI is established when there is insufficient scientific evidence to determine a recommended Dietary Allowance (RDA) (Lim et al., 2020). A wide variety of fruits and vegetables are potassium-rich sources i.e., dried apricots, prunes, bananas, orange juice, potatoes, leafy green vegetables as well as some legumes. Among starchy foods, whole-wheat flour and brown rice are much higher in potassium than their refined counterparts (van der Waal et al., 2012; Wang et al., 2012a). Potassium content in selected food sources is outlined in Fig. 1 (A). The common dietary source of sodium is table salt (sodium chloride), in addition to high sodium foods such as processed foods, which not only contains a significant amount of sodium chloride, but also contains sodium food additives such as monosodium glutamate, and other salts as nitrate, nitrite and citrate (Stoll et al., 2020).

**Table 1:** Potassium and Sodium Adequate Intakes. *The AIs do not apply to individuals with impaired potassium excretion because of medical conditions (i.e., kidney disease) or the use of medications that impair potassium excretion (Oria et al., 2019), National Institutes of Health (NIH), 2021.

	Potassium (mg)				Sodium (mg)			
Age	Male	Female	Pregnancy	Lactation	Male	Female	Pregnancy	Lactation
**Infants**								
Birth to 6 months	400	400			110			
7–12 months	860	860			370			
**Children and adolescents**								
1–3 years	2000	2000			800			
4–8 years	2300	2300			1000			
9–13 years	2500	2300			1200			
14–18 years	3000	2300	2600	2500	1500	1500	1500	1500
**Adults and Elderly**								
19–50 years	3400	2600	2900	2800	1500	1500	1500	1500
51+ years	3400	2600			1500

#### 3.1.2 Sodium and potassium deficiency, excessive intake and interaction with other minerals or medications

Sodium is well-conserved by the body, on the contrary, potassium, is more susceptible to loss through sweat, or with excessive intake of diuretic. In fact, potassium concentration is regulated by adrenal hormones which stimulate the kidneys to excrete excessive amounts through urine (Liang et al., 2011). Clinical data have demonstrated that sodium to potassium ratio is crucial for maintaining normal blood pressure and has a higher impact on blood pressure than sodium or potassium intakes alone (Perez and Chang, 2014). Studies reported that high sodium intake, a condition called hypernatremia, is often associated with hypertension, edema and decreased urine excretion which could eventually lead to renal failure (Farquhar et al., 2015). Hyponatremia on the other hand, is usually characterized by headache, confusion, muscle spasms, nausea and vomiting. However, sodium depletion is rarely a result of dietary sodium deficiency with little evidence for its adverse effects (Medicine, 2006; Reynolds et al., 2006). High potassium level, a condition called hyperkalemia, is characterized by irritability, nausea and altered urine excretion. Severe potassium disorders can lead to life-threatening cardiac conduction disturbances and neuromuscular dysfunction (Ravioli et al., 2021). Chronic potassium deficiency is characterized by muscle weakness and acne or dry skin. These symptoms may progress to nervous disorders, decreased or irregular heartbeat and eventually could lead to loss of gastrointestinal tone if left untreated (Teymoori et al., 2021). Cardiac arrhythmia is one of the most serious complications that happen in severe cases due to the sudden loss of potassium. Deficiency of potassium may also impair glucose metabolism and lead to elevated blood sugar (Pohl et al., 2013).

#### 3.1.3 Sodium and potassium throughout the human life cycle and sex effect

##### 3.1.3.1 Sodium and potassium during pregnancy and lactation

Maternal nutrition is crucial for normal fetal growth during pregnancy and in minimizing child-birth defects (Kermani and Nematy, 2018). At this stage, sodium and potassium intakes are particularly important in maintaining cardiovascular health. The association of sodium and potassium intakes and their ratios with blood pressure in normotensive pregnant women was evaluated in a cross-sectional study. Data demonstrated that dietary sodium and potassium have no effect on blood pressure during pregnancy of normotensive women. Nevertheless, the blood pressure of pregnant women with high risk of preeclampsia was affected by sodium intake. Therefore, the Dietary Approach to Stop Hypertension Diet (DASH) is recommended for women with preeclampsia after delivery to avoid hypertension complications (Lane-Cordova et al., 2019). In accordance with these findings, Yilmaz et al. demonstrated that blood pressure was directly correlated with sodium to potassium ratio in case of pregnant women with preeclampsia. Results showed that not only the dietary ratio of both minerals was associated with a change in systolic blood pressure and creatinine levels, the study also revealed the adverse effects that high sodium, low potassium ratio can have on the renal function. Thus, pregnant women with preeclampsia are encouraged to optimize their sodium to potassium ratio (Yilmaz et al., 2017). Wolak et al. showed that imbalance in serum potassium levels in the early stages of pregnancy increases the risk of preeclampsia and gestational hypertension and diabetes mellitus in later stages of pregnancy (Wolak et al., 2016). Significance of sodium and potassium throughout the human life cycle and sex effect is outlined in Fig. 1 (B).

##### 3.1.3.2 Sodium and potassium for infants and children

Proper nutrition at early stages is critical for infants’ growth and development, thus nutritionists continue to assess nutrient needs for infants and children, which will provide invaluable considerations when designing preschool diets and infant formulas (Merkiel and Chalcarz, 2016; Davidsson, 1994). Merkiel et al. investigated the dietary patterns of selected nutrients including sodium and potassium intake for 128 children (aged between 4 and 6 years old) and found that the average intakes of 71.1% of preschoolers exceeded the tolerable upper limit (UL) of their dietary sodium. In contrast, they had 70% lower than their required AI of potassium. High sodium intake amongst children increases their risk of hypertension, which is also aggravated by the low potassium intake. Moreover, this dietary pattern could increase their risk of bone demineralization and ultimately lead to osteoporosis since high sodium diet increases urinary calcium loss (Merkiel and Chalcarz, 2016). In addition, high sodium intake was demonstrated to have a deleterious effect on insulin sensitivity in children (Teymoori et al., 2021; Park et al., 2018). Notably, obese children tend to have higher sodium and lower potassium intakes than their normal-weight counterparts (Fig. 1 (B)).

##### 3.1.3.3 Sodium and potassium for adults

Adopting a healthy diet DASH (sodium intake should not exceed 2.3 mg per day for adults and 1.5 mg for high-risk populations) would mitigate the risk of many diseases such as hypertension and cardiovascular diseases. A cross-sectional study observed a significant increase in systolic blood pressure with high sodium to potassium ratio in hypertensive and normotensive adults. On the contrary, an insignificant association of high ratio with diastolic blood pressure in hypotensive individuals was observed (Li et al., 2020). One of the most intriguing findings is the relation between high sodium intakes and declined cognitive functions in adults. Many clinical trials emphasized the neurotoxicity of high sodium levels as well as its effect on systemic and cerebral blood vessels. These studies suggest that high sodium diet could lead to reduced blood flow to the brain and ultimately resulting in cognitive impairment (Wenzel et al., 2019). Further clinical trials are required to confirm the link between high sodium intake and cognitive impairment (Mohan et al., 2020). Furthermore, sodium to potassium ratio has been linked to insulin resistance and reduced sensitivity in adults. Park et al. demonstrated that low-sodium diets decreased insulin resistance whereas high-sodium intake increased homeostatic model assessment insulin resistance (HoMA-IR). Despite these findings, further studies are required to confirm the impact of sodium to potassium ratio on insulin sensitivity in adults and other age groups (Fig. 1 (B)), (Park et al., 2018).

##### 3.1.3.4 Sodium and potassium in elderly

High serum sodium levels usually have higher effect on systolic blood pressure especially in the age between 70 and 85 years. Interestingly, serum potassium levels dominate the effect on the systolic blood pressure for individuals under 70 years old. Jung et al. examined the relation between sodium to potassium ratio with arterial stiffness as well as the subclinical atherosclerosis in elderly individuals. The study demonstrated a positive correlation between sodium to potassium ratio to the levels of brachial-ankle pulse wave velocity and aortic intima media thickness, revealing a direct impact on vascular damage (Jung et al., 2019). Nevertheless, a study has attempted to use the variation of serum sodium as a prognosticator of mortality in elderly. However, they were not able to produce a predictive value from the static measures, despite the fact that dynamic measures of dysnatremia were correlated with high risk of mortality (Fig. 1 (B)), (Barma et al., 2018).

##### 3.1.3.5 Gender effect on sodium and potassium

Blood pressure response in relation to dietary sodium and potassium can vary according to age and gender (He et al., 2009). However, the association of high serum potassium and low blood pressure is consistent amongst most age groups. Men usually have higher systolic blood pressure than women, however, after menopause, women's systolic blood pressure can be equivalent to men, and could even surpass that of men by the age of 70 years or above (Maas and Appelman, 2010). The less susceptibility of women to hypertension before menopause could be attributed to the role of estrogen as well as other hormones in reducing hypertension risk (Jeong et al., 2020). Migdal et al. explored the effect of high, low and medium salt diets on central blood pressure revealing that males had a higher systolic and diastolic average real variability than their female counterparts, despite having the exact diets (Migdal et al., 2020). The varied relationship between dietary sodium and potassium with hypertension risk in males and females can be ascribed to their different genetics, especially the variation in the expression of renin-angiotensin components among both genders. Jeong et al. reported that only women with FGF5 rs16998073 wild-type AA allele who consume ≥3500 mg/day are at decreased risk of hypertension compared to their male counterpart, regardless of their sodium to potassium intake ratio Fig. 1 (B) (Jeong et al., 2020).

### 3.2 Calcium and phosphorous

#### 3.2.1 Calcium and phosphorous functions, sources, dietary intake and bioavailability

**Fig. 2:**
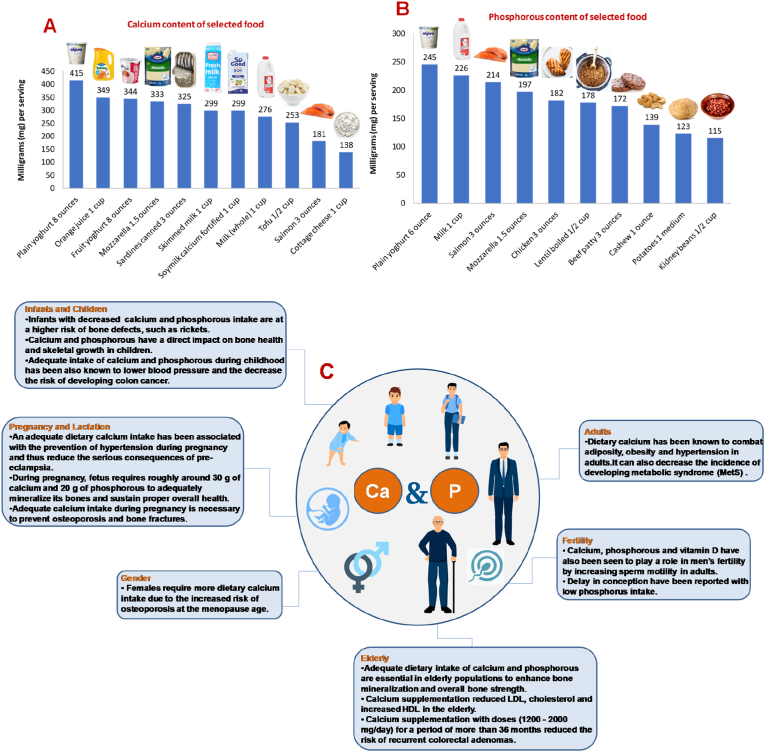
(A) Calcium content of selected food (B) Phosphorous content of selected food (C) Significance of calcium and phosphorous throughout the human life cycle and sex effect.

Calcium and phosphate are pivotal macrominerals required for neuromuscular function and skeletal mineralization (Lombardi et al., 2020). Calcium is one of the most abundant electrolytes in the body which plays a critical role in cell membrane function and intracellular signaling. Almost 99% of total calcium is located in bone and teeth, acting structurally as supporting material in the form of calcium hydroxyapatite [Ca_10_(PO_4_)_6_(OH)_2_] (Peacock, 2010), whereas less than 1% is found in extracellular fluids. The latter fraction is present as an active free ionized form which interacts directly with cell membranes, calcium channels and with calcium sensing receptor (CaSR), hence it determines the physiological effect of calcium (i.e., blood vessel contraction, muscle tone and nerve transmission) (Bushinsky and Monk, 1998; Ross et al., 2011a). Calcium exchange between extracellular fluids and bone is an essential dynamic part of bone remodeling via a rapidly exchangeable pool. Urinary excretion is also involved in achieving the calcium homeostasis (Sun et al., 2020). Similar to calcium, phosphorus is also found predominantly 85% in mineralized bone in the form of hydroxyapatite where only 10% exists in soft tissues, and the remaining 2–3% circulates in the form of inorganic phosphate in extracellular fluids, constituting a phosphate pool that could be exchangeable. Phosphate is an important constituent of bone mineral, and in growing individuals, the balance of phosphate must be positive to meet the needs of skeletal growth (Sun et al., 2020; Yee et al., 2021). Phosphorus is also a predominant component of DNA, RNA, cell membrane structure (in the form of phospholipids) and involved in the main source of energy synthesis, ATP. It is also required for phosphorylation of many proteins and sugars (Sun et al., 2020; Bergman et al., 2009). In terms of homeostasis, these two macrominerals are interconnected and controlled mainly by the interplay between hormones as parathyroid hormone (PTH), fat-soluble vitamin (vitamin D) and fibroblast growth factor (FGF23), which are regulated by three major organ systems, the intestine, kidney and bone (Sun et al., 2020). Calcium-rich foods are mainly dairy products, especially plain yoghurt, mozzarella cheese, and milk. Non-diary calcium sources are also available, as orange juice, nuts, sesame and chia seeds. Vegetables rich in calcium are kale, broccoli and watercress (Baron et al., 1999a). Calcium is also found in some medicines (i.e., antacids) and as a dietary supplement. Lactose intolerant patients must resort to other sources of calcium due to the importance of such mineral. Calcium content of selected food is outlined in Fig. 2 (A). Phosphorous is also found in healthy foods including dairy products, meats and poultry, fish, eggs, nuts, legumes, vegetables and grains (Calvo et al., 2014). However, dairy products contribute for the highest value (20%) of total phosphorus intakes, and to a lesser extent (10%) from bakery products (i.e., breads) while vegetables and chicken only account for 5% each. The rate of phosphorous absorption is higher from animal sources when compared to the plants (Calvo et al., 2014). Phosphate is also present as food additives (i.e., inorganic phosphate) in many processed food products owing to its ability of preserving moisture or stabilizing frozen foods (Leon et al., 2013). Phosphorous content of selected food is outlined in Fig. 2 (B). The recommended values of calcium and phosphorus for adults are 700 mg/d and 550 mg/d respectively (Madeo et al., 2018). The RDA of calcium and phosphorous varies throughout the human life cycle as highlighted in Table 2. RDA is the average daily level of intake sufficient to meet the nutrient requirements of nearly all (97%–98%) healthy individuals; often used to plan nutritionally adequate diets for individuals.

**Table 2:** Recommended dietary allowances (RDA) for calcium and phosphorous. *Adequate Intake (AI) (Bergman et al., 2009), National institutes of health (NIH), 2021.

	Calcium (mg)				Phosphorous (mg)			
Age	Male	Female	Pregnancy	Lactation	Male	Female	Pregnancy	Lactation
**Infants**								
Birth to 6 months*	200	200			100	100		
7–12 months*	260	260			275	275		
**Children and adolescents**								
1–3 years	700	700			460	460		
4–8 years	1000	1000			500	500		
9–13 years	1300	1300			1250	1250		
14–18 years	1300	1300	1300	1300	1250	1250	1250	1250
**Adults and Elderly**								
19–50 years	1000	1000	1000	1000	700	700	700	700
51+ years	1000	1200			700	700		

#### 3.2.2 Calcium and phosphorous deficiency, excessive intake and interaction with other minerals or medications

Calcium deficiency, hypocalcaemia, can reduce bone density leading to osteoporosis, which is characterized by fragile bones. Another medical condition in children, rickets, is also caused by calcium deficiency although these disorders are more commonly accompanied by vitamin D deficiency. Osteomalacia in adults and children, another condition of chronic calcium deficiency, is characterized by defective bone mineralization. In these disorders, the requirements for calcium and vitamin D appear to be interrelated where the lower serum vitamin D level (measured as 25-hydroxyvitamin D), the more calcium is required (Sempos et al., 2021). Hypocalcemia (serum calcium <8.5 mg/dL) is usually a result of a vitamin D deficiency, hypoparathyroidism, impaired bone resorption of calcium or as an adverse effect of certain medications (i.e., bisphosphonates, cisplatin or proton pump inhibitors) (Fong and Khan, 2012; Bove-Fenderson and Mannstadt, 2018; Pepe et al., 2020). On the other hand, hypercalcemia (serum levels >10.5 mg/dL) and hypercalciuria (urinary calcium levels >250 mg/day) were rarely reported in healthy individuals, however, it could result from cancer and primary hyperparathyroidism (Cormick et al., 2015). In fact, hypercalcemia and hypercalciuria symptoms can cause poor muscle tone, renal insufficiency, gastrointestinal symptoms as constipation, nausea, weight loss, fatigue and heart arrhythmias (Cormick et al., 2021). Calcium supplements are commonly used to prevent fracture in postmenopausal women. However, whether these supplements increase the risk of kidney stone formation is still controversial. Recent findings showed that a dose of 500 mg of calcium plus 200 units of vitamin D increased risk of renal stones and could also affect iron absorption (Cormick and Belizán, 2019). Hypophosphatemia (phosphorus deficiency) is considered a rare medical condition. Symptoms could include anorexia, anemia, proximal muscle weakness, skeletal effects as bone pain, rickets and osteomalacia. Hypophosphatemia is also associated with hyperparathyroidism, renal impairment and diabetic ketoacidosis (Calvo and Uribarri, 2013). On the other hand, high phosphorus intakes (1000 mg/day) may be associated with cardiovascular, kidney and bone diseases, however these adverse effects were rarely reported (Chang et al., 2017). Regarding phosphorous interactions with medications, it was reported that antacids decrease the intestinal absorption of dietary phosphorous, whereas laxatives containing sodium phosphate could increase serum phosphate levels (Casais et al., 2009). Considering that old aged groups are more to administer laxative and antacid drugs, special consideration of such interaction ought to be considered.

#### 3.2.3 Calcium and phosphorous throughout the human life cycle and sex effect

##### 3.2.3.1 Calcium and phosphorous during pregnancy and lactation

An adequate dietary calcium intake has been associated not only with the prevention of hypertension during pregnancy but also with the reduction of low-density lipoprotein (LDL), cholesterol levels and prevention of osteoporosis and colorectal adenomas (Cormick and Belizan, 2019). However, the benefits of calcium supplementation on bone health seem to be greater in children and adolescents with higher needs of calcium intake (Oliai Araghi et al., 2020). Owing to the serious consequences of hypertension in pregnancy, several studies have targeted to decrease the development of pre-eclampsia in pregnant women during their first couple of weeks of pregnancy. Calcium supplementation in the second half of pregnancy is known to reduce the detrimental effects of pre-eclampsia; however, the effect of calcium supplementation during placentation is not known (Hofmeyr et al., 2019). DeSousa et al. demonstrated that calcium supplementation prevented endothelial cell activation induced by trophoblastic debris from preeclamptic placentae. These findings may suggest the protective mechanism of calcium supplementation on preeclampsia (DeSousa et al., 2016). The WHO recommends that pregnant women be provided calcium supplements (1.5–2 g/day calcium) to prevent preeclampsia, especially in sectors of the population which exhibit inadequate calcium intake. This is the first recommended nutritional intervention to prevent this condition, a leading cause of maternal mortality globally (Newmark et al., 2004; Omotayo et al., 2016). During pregnancy, the amount of absorbed intestinal calcium increases two folds via vitamin D, in order to adequately supply enough of this mineral for the fetus. In this context, during pregnancy, a fetus requires roughly around 30 g of calcium and 20 g of phosphorous to adequately mineralize its bones and sustain proper overall health. Changes in mineral metabolism initiated during pregnancy as well as a decrease in the levels of dietary calcium as well as vitamin D may also cause bone resorption (Kovacs and Ralston, 2015). Therefore, adequate calcium intake during pregnancy is necessary to avoid bone fractures, though nonetheless, no long-term risks of osteoporosis or fracture have been recorded (Kovacs, 2018). Other studies showed that women who get pregnant with previous optimal intakes may not require excessive amounts of calcium to accommodate for the fetal needs (Hacker et al., 2012). Significance of calcium and phosphorous throughout the human life cycle and sex effect is outlined in Fig. 2 (C).

##### 3.2.3.2 Calcium and phosphorous for infants and children

Phosphorous and calcium play critical role in the prevention of detrimental bone diseases during the infant stage. Infants less than 6 months old are required to intake 200–1000 mg/day of calcium along with vitamin D (400 IU/day), while infants from 6 months to one year of age should receive 260–1500 mg/day. Infants who exhibit a decrease in nutritional intake of key minerals are at a higher risk of bone defects as rickets (Holick, 2006; Ross et al., 2011b). In children, the skeletal effects of calcium deficiency may be exacerbated with greater imbalance in calcium to phosphorus ratio. Dietary calcium and phosphorous supplementation should increase with age to effectively attain skeletal growth. The RDA of phosphorous in toddlers is 460–500 mg, while the mean calcium intake is around 821 mg. Kruger et al. depicted the effect of regular calcium intake on height, weight, whole body bone mineral density of children. Results showed that children receiving regular milk were consequently taller, weighed more and had greater lean mass (Kruger et al., 2017). Skinner et al. assessed the correlation of dietary calcium intake to children's body fat. Fifty-two children at 8 years of age of similar BMIs were assessed. Dietary calcium as well as polyunsaturated fat intake was negatively correlated to the percent of body fat (Skinner et al., 2003). This finding depicts the importance of inclusion of calcium-rich foods in a child's diet to actively maintain a healthy lifestyle. Adequate calcium intake during childhood has been also known to lower blood pressure 75 and the decrease the risk of developing colon cancer (Fig. 2 (C)), (Baron et al., 1999b).

##### 3.2.3.3 Calcium and phosphorous for adults

Calcium intake throughout our lifetime remains vital (Cormick and Belizán, 2019; Heaney, 2006). Adults are also recommended to maintain an RDA of 700 mg of phosphorous. In fact, adults who extensively consume dairy products are more likely to have higher phosphorous density values (mg/kcal). This is due to the increase in phosphorous density of cow milk (I. o. M. S. C. o. t. S. E. o. D. R. Intakes, 1997). Dietary calcium has been known to combat adiposity, obesity and hypertension in adults. Whereas Cormick et al. showed that patients who received higher calcium intake (>800 mg/d) had lower prevalence of obesity when compared to low calcium intake group (<800 mg/d) (Cormick and Belizán, 2019). Recent data also showed the decreased incidence of developing metabolic syndrome (MetS) upon dietary calcium intake (300 mg/day) (Woo et al., 2020). Regarding the significance of calcium and phosphorous during fertility, it was observed that calcium, supplied from the maternal decidua, plays a role in egg fertilization and blastocyst implantation. The levels of calcium, which is supplied to the offspring from the mother, increases over time throughout the stages of pregnancy (Kovacs, 2018). Calcium and vitamin D have also been seen to play a role in men's fertility by increasing sperm motility in adult population. One study showed a positive correlation between the levels of serum calcium and sperm motility 80. Furthermore, delay in conception have been reported with low phosphorus intake (Fig. 2 (C)), (Richardson, 1997).

##### *3.2.3.4* Calcium and phosphorous *in elderly*

Calcium and phosphorous requirements vary with the progression in age amongst individuals. The role of calcium on the retinal function was assessed on adults aged 50 years and above. Results showed that lower intake of dairy products increased the incidence of visual disorders (Gopinath et al., 2014a). Decrease of calcium and phosphorous levels has also been known to alter bone mineral density in the elderly. In fact, adequate dietary intake of calcium and phosphorous are essential in elderly populations to enhance bone mineralization and overall bone strength (Cormick and Belizán, 2019). Kiehn et al. depicted a positive correlation between the low dietary intake of calcium and vitamin D and increased frequency of bone fractures in elderly (Kiehn et al., 2009). Recent findings of calcium supplementation and lipid metabolism showed that calcium supplementation reduced LDL and increased high-density lipoproteins (HDL) in the elderly (Hibler et al., 2020). Similarly, randomized clinical trials reported that calcium supplementation with doses (1200–2000 mg/day) for a period of more than 36 months reduced the risk of recurrent colorectal adenomas (Fig. 2 (C)), (Shah et al., 2020).

##### *3.2.3.5* *Gender effect on* calcium and phosphorous

Adequate dietary calcium is vital to maintain proper skeletal function, substantially maintain bone health specifically in women at the menopause age. Females require more dietary calcium intake due to the increased risk of osteoporosis (Cormick and Belizán, 2019). Studies showed that the calcium requirement in men is even less than that which was previously thought to be 86. Braun et al. also showed that boys retain more calcium than girls after receiving calcium via beverage fortified with calcium citrate malate (Braun et al., 2006). Moreover, calcium intake was shown to be inversely related to blood pressure in girls over the age of five (Fig. 2 (C)), (Gopinath et al., 2014b).

### 3.3 Magnesium

#### 3.3.1 Magnesium functions, sources, dietary intake and bioavailability

**Fig. 3:**
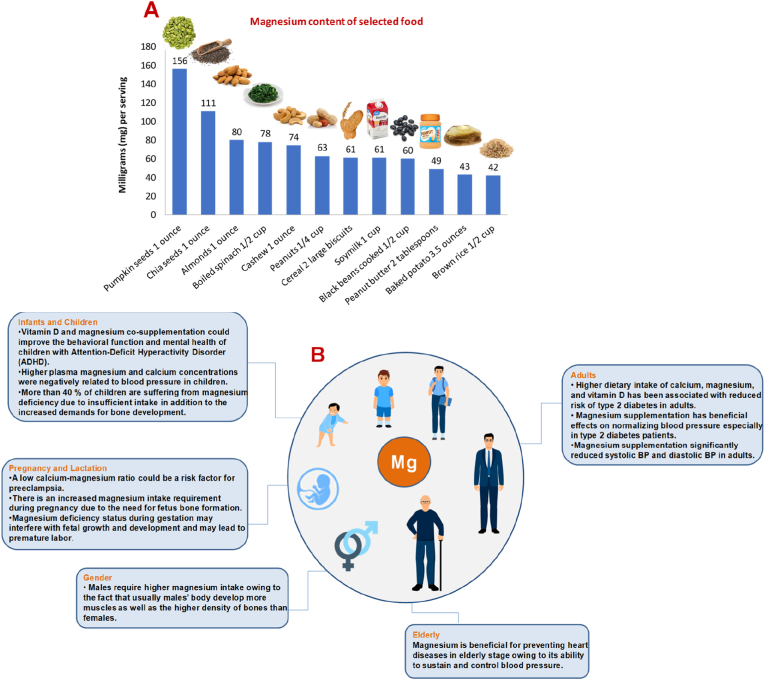
(A) Magnesium content of selected food (B) Significance of magnesium throughout the human life cycle and sex effect.

Magnesium is the fourth most abundant mineral in the body after calcium, potassium, and sodium and the second most abundant cation within the body's cells after potassium. Approximately 99% of total body magnesium is located in bone, muscles and non-muscular soft tissue. Less than 1% of total magnesium is in blood serum. Normal serum magnesium concentrations range between 0.75 and 0.95 mmol (mmol)/L. Magnesium has been recognized as a cofactor in more than 300 enzyme systems that regulate diverse biochemical reactions in the body. Magnesium is also crucial for energy production (ATP), oxidative phosphorylation, and glycolysis. Magnesium also plays a key role in the active transport of calcium and potassium ions across cell membranes, a process that is important to nerve impulse conduction, muscle contraction, vasomotor tone and normal heart rhythm (Grober et al., 2015; Firouzi et al., 2015). Magnesium is found naturally in many sources of health diet, added to other fortified products, also available as a dietary supplement and is a primary ingredient in some medications (i.e., antacids and laxatives) (Guerrera et al., 2009). Green leafy vegetables (i.e., spinach), legumes, nuts, seeds, and whole grains, are some of the rich sources. Breakfast cereals and other fortified foods are also good sources of magnesium. Magnesium content of selected food is outlined in Fig. 3 (A). Magnesium homeostasis is largely regulated by the kidney, which excretes around 120 mg magnesium into the urine per day. Urinary excretion is reduced when magnesium status is low (Grober et al., 2015). The RDA of magnesium throughout the human lifecycle is highlighted in Table 3.

**Table 3:** Recommended dietary allowances (RDAs) for magnesium. *Adequate Intake (AI), National institutes of health (NIH), 2021

	Magnesium (mg)			
Age	Male	Female	Pregnancy	Lactation
**Infants**				
Birth to 6 months	30*	30*		
7–12 months	75*	75*		
**Children and adolescents**				
1–3 years	80	80		
4–8 years	130	130		
9–13 years	240	240		
14–18 years	410	360	400	360
**Adults and Elderly**				
19–30	400	310	350	310
31–50 years	420	320	360	320
51+ years	420	320		

#### 3.3.2 Magnesium deficiency, excessive intake and interaction with other minerals or medications

Magnesium deficiency (<0.75 mmol/L) is rare since the kidneys limit urinary excretion of this mineral in cases of low magnesium intake. Nevertheless, in severe cases where there are extremely low intakes or excessive losses of magnesium due to certain medical conditions as chronic alcoholism or the use of certain medications can lead to magnesium deficiency. Low levels of magnesium (<252 mg/day) have been associated with a number of chronic disorders as Alzheimer's disease, asthma, attention deficit hyperactivity disorder (ADHD), insulin resistance, hypertension, migraine headaches and osteoporosis. In addition, magnesium deficiency exacerbates potassium mediated arrhythmia. Since there is a correlation between magnesium, calcium and potassium, severe magnesium deficiency can result in hypocalcemia or hypokalemia which consequently lead to disruption of minerals homeostasis (Grober et al., 2015). On the other hand, excessive magnesium intake does not pose a health risk in healthy individuals since the excess amounts will be urinary excreted by the kidneys (Musso, 2009). Although magnesium supplements are well tolerated but overdoses of magnesium from medications or dietary supplements could lead to diarrhea that can be associated with nausea and abdominal cramping. The diarrhea and laxative effects of magnesium salts are due to the osmotic activity of unabsorbed salts in the intestine and colon and the stimulation of gastric motility (Fairley et al., 2017). Magnesium intoxication occurs very seldom in human and was only reported with very high doses of magnesium-containing laxatives and antacids (>5000 mg/day) (Onishi and Yoshino, 2006) or in cases of impaired renal function or kidney failure (Musso, 2009). Therefore, attention should be paid in patients with renal insufficiency.

#### 3.3.3 Magnesium throughout the human life cycle and sex effect

##### 3.3.3.1 Magnesium during pregnancy and lactation

Accumulating evidence shows that there is an increased magnesium intake requirement during pregnancy due to the need for fetus bone formation (Fanni et al., 2021). Moreover, Winarno et al. showed that patients with preeclampsia had significantly lower serum calcium-magnesium ratios than did healthy pregnant women; therefore, magnesium deficiency status during gestation may interfere with fetal growth and development and may lead to premature labor (Winarno et al., 2021). Other clinical trials have associated intrauterine growth restriction (IUGR) with a higher risk of undergoing insulin resistance later in life, suggesting that chronic intrauterine magnesium deficiency might result in IUGR. In support to this hypothesis, severe magnesium deficiency in pregnant women might lead to insulin resistance in newborns, with important consequences after birth, ending with the possibility of metabolic syndrome in childhood or adulthood (Fanni et al., 2021). Significance of magnesium throughout the human life cycle and sex effect is outlined in Fig. 3 (B).

##### 3.3.3.2 Magnesium for infants and children

Magnesium is vital for absorption of other macronutrients as calcium by converting vitamin D to its active form, which is indispensable for the development and growth of bones in infants and children (Vormann, 2003). Hemamy et al. showed that vitamin D (50,000 IU/week) and magnesium (6 mg/kg/day) co-supplementation for a duration of 8-weeks could improve the behavioral function and mental health of children with Attention-Deficit Hyperactivity Disorder (ADHD) (Hemamy et al., 2021). Another interesting finding showed that that higher plasma magnesium and calcium concentrations were negatively related to blood pressure in children aged 6–9 years (Chen et al., 2021). It is estimated that more than 40% of children are suffering from magnesium deficiency due to insufficient intake in addition to the increased demands for bone development (Fig. 3 (B)), (Vormann, 2003).

##### 3.3.3.3 Magnesium for adults

Higher dietary intake of calcium, magnesium, and vitamin D has been associated with reduced risk of type 2 diabetes in adults (Shah et al., 2021). A study suggested that magnesium supplementation for >12 weeks, in doses higher than 300 mg/day could significantly decrease both systolic and diastolic blood pressure in type 2 diabetes patients. In support of these findings, Zhang et al. showed that magnesium supplementation at a dose of 368 mg/d for 3 months significantly reduced systolic BP by 2.00 mm Hg and diastolic BP by 1.78 mm Hg. Based on these findings, magnesium supplementation has beneficial effects on normalizing blood pressure especially in type 2 diabetes patients. However, further investigations are needed to provide more reliable evidences (Fig. 3 (B)), (Askari et al., 2021).

##### 3.3.3.4 Magnesium in elderly

Although magnesium is highly needed during the adolescence stage for developing and maintaining healthy bones and muscles, during the elderly stage a high dose of magnesium is also required for preventing heart diseases owing to its ability to sustain and control blood pressure (Zhang et al., 2016a). Vianello et al. investigated magnesium and calcium total circulating levels and the associated pro-inflammatory mediators in elderly acute aortic dissection (AAD) patients, revealing that low magnesium and calcium in AAD elderly patients may contribute to altering normal endothelial physiology and also concur in changing the normal concentrations of different mediators involved in vasodilatation and constriction (Vianello et al., 2017). Therefore, one can conclude that magnesium might be beneficial for preventing heart diseases in elderly stage (Fig. 3 (B)).

##### 3.3.3.5 Gendereffect on magnesium

Recent findings suggest a lower magnesium requirement for healthy men and women than estimated previously (Hunt and Johnson, 2006). Adults require magnesium intake from 360 mg and up to 400 mg per day for males, and from 300 mg up to 320 for females (Nielsen, 2016). In fact, males require higher magnesium intake owing to the fact that usually males’ body develop more muscles as well as the higher density of bones than females (Fig. 3 (B)), (Verkaik-Kloosterman et al., 2012).

### 3.4 Sulfur

#### 3.4.1 Sulfur functions, sources, dietary intake and bioavailability

**Fig. 4:**
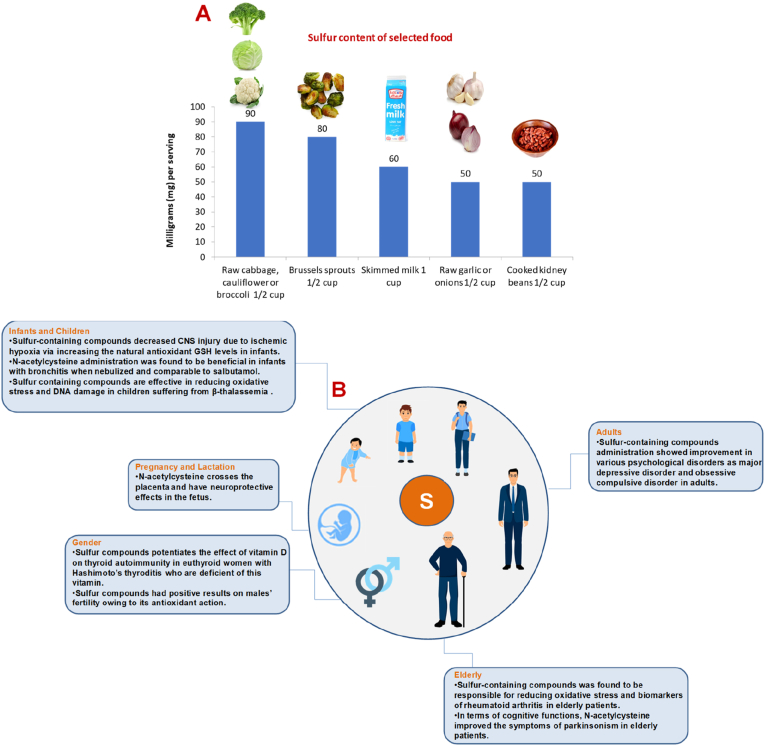
(A) Sulfur content of selected food (B) Significance of sulfur throughout the human life cycle and sex effect.

Sulfur is the third most abundant mineral found in our body, after calcium and phosphorus, and the sixth most abundant macromineral in breast milk. Sulfur has potential beneficial effects on a variety of dermatological disorders (i.e., acne and skin lesions caused by UV) and is added as an active ingredient in acne ointments (Del Rosso, 2009), in antidandruff shampoos and as an antidote for acute exposure to radioactive material (Breneman and Ariano, 1993). Sulfur aids in wound healing and has a history of folk usage as a remedy for skin rashes (Liu et al., 2020). Sulfur is needed for a number of chemical reactions involved in the metabolism of drugs, steroids and xenobiotics. For instance, sulfation is a major pathway for detoxificication of pharmacological agents by the liver (i.e., acetaminophen) (Kane et al., 1995; Pang et al., 1995). Accumulating evidence also suggests that this element plays a role in the synthesis of a very large number of key metabolic intermediates as glutathione (GSH), a natural antioxidant in the body (Miekus et al., 2020). Health benefits of the cruciferous vegetables are mainly due to the presence of sulfur-containing glycosides (glucosinolates, GSLs). In fact, dietary GSLs are known for their anticancer potential (Chang et al., 2010; Modem et al., 2012). A study reported that the risk of colon cancer is enhanced among individuals with a low consumption of cruciferous vegetables (i.e., broccoli and cabbage), and reduced among those with a high consumption of these vegetables. In addition, diet rich in sulfur containing compounds could inhibit the development of inflammation by regulating the pro-inflammatory signaling pathway (i.e., IL-1β, TNFα, and IL-6), stimulating natural defense mechanisms as well as regulating nuclear factor kappa B (NF-kB), which plays an important role in the immune system (Miekus et al., 2020; Hahm and Singh, 2014). Recently, more attention has been given for the use of sulfur-containing compounds in SARS-CoV-2 treatment (Ghidoli et al., 2021; Yamaguchi and Kumagai, 2020; Wang et al., 2012b; Lawson and Hunsaker, 2018). Dietary sulfur is derived almost exclusively from proteins, however only two of the twenty amino acids normally present in proteins contain sulfur and are called sulfur-containing amino acids (SAAs). Of these SAA, methionine, cannot be synthesized by our bodies and thus has to be supplied by the diet. Cysteine, another SAA, can be synthesized by our body, yet it requires a steady supply of sulfur. Both SAAs are required for a large number of key metabolic intermediates essential for life (Nimni et al., 2007). To our knowledge, there is no RDA for sulfur and supplementation that contains sulfur in a form other than SAA is not available. On the other hand, an RDA to methionine and cysteine have been studied since they are representative to sulfur (Nimni et al., 2007). Fruits and vegetables are among the sulfur-rich sources with beneficial effects on human health. In particular, cruciferous vegetables including broccoli, cabbage, cauliflower and other plants of the family Brassicaceae are rich sources of sulfur (Miekus et al., 2020). Onion (Allium cepa) and garlic (Allium sativa) are some of the oldest cultivated plants in the world and are rich sources of natural organosulfur compounds (OSCs) (Farag et al., 2017a, 2017b). Other sulfur-rich sources include brussels sprouts, dairy products, fish, legumes, meats, nuts, raspberries and wheat germ (Marcus and Marcus, 2013). Organic sulfur complexes, notably the amino acids methionine and cysteine, largely meet the sulfur needs of the body. SAA are more abundant in animal and cereal proteins than in legume proteins, with the ratio of methionine to cysteine tending to be higher in animal proteins than in plant sources (Blachier et al., 2020). Dietary SAA analysis and protein supplementation may be indicated for vegan athletes, children, or patients with HIV due to the increased risk of SAA deficiency in these groups. Sulfur content of selected food is outlined in Fig. 4 (A).

#### 3.4.2 Sulfur deficiency, excessive intake and interaction with other minerals or medications

There are no risks of sulfur deficiency, unless there is extreme protein deprivation, or toxicity, unless protein supplements with sulfur are excessively consumed (Marcus and Marcus, 2013). This is mainly due to the fact that neither cysteine nor methionine are stored in the body. Thus, any dietary excess is readily oxidized to sulfate, excreted in urine or stored in the form of GSH (Nimni et al., 2007). Similarly, under conditions of low SAA intake, protein synthesis will be maintained whereas the synthesis of sulfate and GSH will be curtailed. Changes in the availability of GSH are likely to negatively influence the function of the immune system and affect the antioxidant defense mechanisms of the body (Nimni et al., 2007).

#### 3.4.3 Sulfur throughout the human life cycle and sex effect

##### 3.4.3.1 Sulfur during pregnancy and lactation

Sulfur-containing compounds were found to have beneficial effects in some of the complications associated with pregnancy. Zhang et al. studied the effect of sulfur containing compounds on intrahepatic cholestasis during pregnancy. Findings showed that symptoms, including pruritus, are improved and liver enzymes were reduced as well (Zhang et al., 2015, 2016b). Further studies were conducted to assess the potential of sulfur-containing compounds in treating some medical conditions of fetus when administered to the pregnant mother. Wiest et al. suggested that N-acetylcysteine crosses the placenta in a rapid rate assuming that it can freely reach the fetus circulation and have protective effects on the fetus neurons against potential neuroinflammation. In support of these findings, further studies were performed to evaluate the clinical safety of antenatal and postnatal N-acetylcysteine as a neuroprotective agent in maternal chorioamnionitis. Findings showed a positive relation between N-acetylcysteine administration to pregnant mothers and its neuroprotective effects in the fetus. In fact, newborns exposed to chorioamnionitis, antenatal and postnatal N-acetylcysteine were safe, preserved cerebrovascular regulation, and increased an anti-inflammatory neuroprotective protein (Wiest et al., 2014). Significance of sulfur throughout the human life cycle and sex effect is outlined in Fig. 4 (B).

##### 3.4.3.2 Sulfur for infants and children

Infants receive their needs from sulfur exclusively from breast milk protein or formula. Nonetheless, some research suggests a potential effect of sulfur containing compounds on some diseases. Moss et al. investigated the effect of N-acetylcysteine administration on GSH levels in infants with CNS injury due to ischemic hypoxia. This injury induces an oxidative stress in the brain that could be alleviated with the natural antioxidant GSH. Findings revealed that administration N-acetylcysteine significantly increased the levels of GSH (Moss et al., 2018). In addition, N-acetylcysteine administration was found to be beneficial in infants with bronchitis when nebulized and comparable to salbutamol (Naz et al., 2014). Evidence suggests that sulfur containing compounds are effective in reducing oxidative stress and DNA damage in children suffering from β-thalassemia (Ozdemir et al., 2014). On the other contrary, no signs of improvements were observed with children with autism when given N-acetylcysteine even though this disorder is highly linked to oxidative stress factors (Fig. 4 (B)), (Dean et al., 2017).

##### 3.4.3.3 Sulfur for adults

Lete et al. investigated the effectiveness of N-acetylcysteine with alpha lipoic acid and bromelain in one antioxidant formula in the treatment of endometriosis-associated pelvic pain in adult women. Results indicated that this formula relieved the pain and reduced the need to take analgesics. However, one of the drawbacks of this study is its inability to identify the effect of each compound separately (Lete et al., 2018). Regarding psychological disorders, Cullen et al. suggested that sulfur-containing compounds administration showed improvement in various psychological disorders in adults (Cullen et al., 2018). This was further supported by Afshar et al. who found that N-acetylcysteine to be effective in obsessive compulsive disorder as an adjuvant therapy (Afshar et al., 2012). In addition, Targum et al. concluded that S-adenosylmethionine enhances the symptoms of major depressive disorder and was further supported by Sarris et al. (Fig. 4 (B)), (Sarris et al., 2018).

##### 3.4.3.4 Sulfur in elderly

The antioxidant effect of sulfur-containing compounds was studied by Hashemi et al. and was found to be responsible for reducing oxidative stress and biomarkers of rheumatoid arthritis in elderly patients (Hashemi et al., 2019). In terms of cognitive functions, Monte et al. managed to relate N-acetylcysteine to dopaminergic pathway in elderly Parkinson patients by increasing this neurotransmitter and consequently improving the symptoms (Fig. 4 (B)), (Monti et al., 2016).

##### 3.4.3.5 Gendereffect on sulfur

Findings suggested that selenomethionine potentiates the effect of vitamin D on thyroid autoimmunity in euthyroid women with Hashimoto's thyroditis who are deficient of this vitamin (Krysiak et al., 2019). Regarding adult males, Dattilo et al. tested a formula containing sulfur compounds on males' fertility with unknown reason. The study found positive results manifested by increasing pregnancy rates and the author related this to its antioxidant action (Fig. 4 (B)), (Dattilo et al., 2014).

## 4 Conclusions and future perspectives

Macrominerals play a significant role in a wide variety of metabolic and physiologic processes in the human body, as well as their well-known importance for proper functioning organs and overall good development and growth of our bodies. In fact, most of the biochemical reactions are dependent on or affected by these electrolytes. Their roles in wellness and disease are dynamic and controversial. The current review aimed at identifying the role of macrominerals as calcium, phosphorus, magnesium, sodium, potassium and sulfur in human health, in addition to their absorption and homeostasis in the body. Here in, we also focused on the critical importance of a well-balanced macromineral intake and their significance throughout the human life cycle. We also demonstrated that dietary requirements can vary according to age and gender. One of the most intriguing macrominerals in the current review that indeed merit further investigation is sulfur. Although macrominerals are quite known for their significance to our health, sulfur is one of the most under estimated minerals that need more focus to further assess the mechanism of its health benefits. The low toxicological profiles of these sulfur compounds, combined with promising therapeutic effects, warrant continued human clinical trials. Fruits and vegetables carry vibrant and indispensable amounts of macrominerals which are fundamental to disease prevention. However, with the increase in consumption of processed foods, macrominerals intake was dramatically affected in large numbers of populations. We propose that healthy diet containing adequate amounts of these macrominerals might have a great potential of preventing or mitigating against various medical conditions. Macrominerals-rich food sources and the recommended daily intake of each macromineral were discussed herein. Recently, great attention has been given for identifying the recommended daily intake of each macromineral since a number of health risks were associated with either their deficiency or excessive intake. In this review, we further highlighted the health risks associated with the decrease or increase in their levels. Future studies should aim at expanding our knowledge for using these macrominerals in the prevention or treatment of many disorders, systematic review for each mineral should be the next logical step for their role in each life cycle to be conclusive. Based on the in-depth understanding of the factors influencing the metabolism of macrominerals, we could better explore their safety and possible therapeutic potential in specific disorders. In this context, it would also be worthwhile to further investigate the metabolites of macrominerals in serum or urine via - omics technology, especially proteomics and metabolomics. Additionally, it is demonstrated that there is still a need to precisely demonstrate the bioavailability of macrominerals from various types of functional food. Finally, the broad spectrum of interactions between different macrominerals or macrominerals with certain drugs still merits further investigations. In fact, these interactions could alter the absorption of dietary macrominerals and hence affects its significance to the human health. Once we better understand how these macrominerals work together, we can design therapeutic regimens that maximize their benefits.

## CRediT authorship contribution statement

**Mohamed A. Farag:** Writing – original draft. **Bishoy Abib:** Writing – original draft, Data curation, Investigation. **Zhiwei Qin:** Writing – review & editing. **Xiaolei Ze:** Writing – review & editing. **Sara E. Ali:** Writing – original draft, Data curation, Investigation, Methodology.

## Declaration of competing interest

The authors declare that they have no known competing financial interests or personal relationships that could have appeared to influence the work reported in this paper.

## References

[bib1] Afshar H., Roohafza H., Mohammad-Beigi H., Haghighi M., Jahangard L., Shokouh P., Sadeghi M., Hafezian H. (2012). N-acetylcysteine add-on treatment in refractory obsessive-compulsive disorder: a randomized, double-blind, placebo-controlled trial. J. Clin. Psychopharmacol..

[bib3] Askari M., Mozaffari H., Jafari A., Ghanbari M., Darooghegi Mofrad M. (2021). The effects of magnesium supplementation on obesity measures in adults: a systematic review and dose-response meta-analysis of randomized controlled trials. Crit. Rev. Food Sci. Nutr..

[bib4] Barma M.A., Soiza R.L., Donnan P.T., McGilchrist M.M., Frost H., Witham M.D. (2018). Serum sodium level variability as a prognosticator in older adults. Scand. J. Clin. Lab. Invest..

[bib5] Baron J.A., Beach M., Mandel J.S., van Stolk R.U., Haile R.W., Sandler R.S., Rothstein R., Summers R.W., Snover D.C., Beck G.J., Bond J.H., Greenberg E.R. (1999). Calcium supplements for the prevention of colorectal adenomas. Calcium polyp prevention study group. N. Engl. J. Med..

[bib6] Baron J., Beach M.f., Mandel J., Van Stolk R., Haile R., Sandler R., Rothstein R., Summers R., Snover D., Beck G. (1999). Calcium supplements for the prevention of colorectal adenomas. N. Engl. J. Med..

[bib7] Bergman C., Gray-Scott D., Chen J.J., Meacham S. (2009). What is next for the Dietary Reference Intakes for bone metabolism related nutrients beyond calcium: phosphorus, magnesium, vitamin D, and fluoride?. Crit. Rev. Food Sci. Nutr..

[bib8] Blachier F., Andriamihaja M., Blais A. (2020). Sulfur-containing amino acids and lipid metabolism. J. Nutr..

[bib9] Bove-Fenderson E., Mannstadt M. (2018). Hypocalcemic disorders, Best practice & research. Clinical endocrinology & metabolism.

[bib10] Braun M., Martin B.R., Kern M., McCabe G.P., Peacock M., Jiang Z., Weaver C.M. (2006). Calcium retention in adolescent boys on a range of controlled calcium intakes. Am. J. Clin. Nutr..

[bib11] Breneman D.L., Ariano M.C. (1993). Successful treatment of acne vulgaris in women with a new topical sodium sulfacetamide/sulfur lotion. Int. J. Dermatol..

[bib12] Bushinsky D.A., Monk R.D. (1998). Electrolyte quintet: calcium. Lancet.

[bib13] Calvo M.S., Uribarri J. (2013). Public health impact of dietary phosphorus excess on bone and cardiovascular health in the general population. Am. J. Clin. Nutr..

[bib14] Calvo M.S., Moshfegh A.J., Tucker K.L. (2014). Assessing the health impact of phosphorus in the food supply: issues and considerations. Adv. Nutr..

[bib15] Casais M.N., Rosa-Diez G., Perez S., Mansilla E.N., Bravo S., Bonofiglio F.C. (2009). Hyperphosphatemia after sodium phosphate laxatives in low risk patients: prospective study. World J. Gastroenterol..

[bib16] Chang H.S., Ko M., Ishizuka M., Fujita S., Yabuki A., Hossain M.A., Yamato O. (2010). Sodium 2-propenyl thiosulfate derived from garlic induces phase II detoxification enzymes in rat hepatoma H4IIE cells. Nutr. Res..

[bib17] Chang A.R., Miller E.R., Anderson C.A., Juraschek S.P., Moser M., White K., Henry B., Krekel C., Oh S., Charleston J., Appel L.J. (2017). Phosphorus additives and albuminuria in early stages of CKD: a randomized controlled trial. Am. J. Kidney Dis. : the official journal of the National Kidney Foundation.

[bib18] Chen G., Li Y., Deng G., Shrestha S., Chen F., Wei Y., Huang Z., Pan J., Zhang Z. (2021). Associations of plasma copper, magnesium, and calcium levels with blood pressure in children: a cross-sectional study. Biol. Trace Elem. Res..

[bib19] Cormick G., Belizán J.M. (2019). Calcium intake and health. Nutrients.

[bib20] Cormick G., Belizan J.M. (2019). Calcium intake and health. Nutrients.

[bib21] Cormick G., Ciapponi A., Cafferata M.L., Belizan J.M. (2015). Calcium supplementation for prevention of primary hypertension. Cochrane Database Syst. Rev..

[bib22] Cormick G., Ciapponi A., Cafferata M.L., Cormick M.S., Belizan J.M. (2021). Calcium supplementation for prevention of primary hypertension. Cochrane Database Syst. Rev..

[bib23] Cullen K.R., Klimes-Dougan B., Westlund Schreiner M., Carstedt P., Marka N., Nelson K., Miller M.J., Reigstad K., Westervelt A., Gunlicks-Stoessel M., Eberly L.E. (2018). N-acetylcysteine for nonsuicidal self-injurious behavior in adolescents: an open-label pilot study. J. Child Adolesc. Psychopharmacol..

[bib24] Dattilo M., Cornet D., Amar E., Cohen M., Menezo Y. (2014). The importance of the one carbon cycle nutritional support in human male fertility: a preliminary clinical report. Reprod. Biol. Endocrinol. : RBE (Rev. Bras. Entomol.).

[bib25] Davidsson L. (1994). Minerals and trace-elements in infant nutrition. Acta Paediatr..

[bib26] Dean O.M., Gray K.M., Villagonzalo K.A., Dodd S., Mohebbi M., Vick T., Tonge B.J., Berk M. (2017). A randomised, double blind, placebo-controlled trial of a fixed dose of N-acetyl cysteine in children with autistic disorder. Aust. N. Z. J. Psychiatr..

[bib27] Del Rosso J.Q. (2009). The use of sodium sulfacetamide 10%-sulfur 5% emollient foam in the treatment of acne vulgaris. The Journal of clinical and aesthetic dermatology.

[bib28] DeSousa J., Tong M., Wei J., Chamley L., Stone P., Chen Q. (2016). The anti-inflammatory effect of calcium for preventing endothelial cell activation in preeclampsia. J. Hum. Hypertens..

[bib30] Fairley J.L., Zhang L., Glassford N.J., Bellomo R. (2017). Magnesium status and magnesium therapy in cardiac surgery: a systematic review and meta-analysis focusing on arrhythmia prevention. J. Crit. Care.

[bib31] Fanni D., Gerosa C., Nurchi V.M., Manchia M., Saba L., Coghe F., Crisponi G., Gibo Y., Van Eyken P., Fanos V., Faa G. (2021). The role of magnesium in pregnancy and in fetal programming of adult diseases. Biol. Trace Elem. Res..

[bib32] Farag M.A., Ali S.E., Hodaya R.H., El-Seedi H.R., Sultani H.N., Laub A., Eissa T.F., Abou-Zaid F.O.F., Wessjohann L.A. (2017). Phytochemical profiles and antimicrobial activities of Allium cepa red cv. and A. Sativum subjected to different drying methods: a comparative MS-based metabolomics. Molecules.

[bib33] Farag M.A., Ali S.E., Hodaya R.H., El-Seedi H.R., Sultani H.N., Laub A., Eissa T.F., Abou-Zaid F.O.F., Wessjohann L.A. (2017). Phytochemical profiles and antimicrobial activities of Allium cepa red cv. and A. Sativum subjected to different drying methods: a comparative MS-based metabolomics. Molecules.

[bib34] Farquhar W.B., Edwards D.G., Jurkovitz C.T., Weintraub W.S. (2015). Dietary sodium and health: more than just blood pressure. J. Am. Coll. Cardiol..

[bib35] Firouzi A., Maadani M., Kiani R., Shakerian F., Sanati H.R., Zahedmehr A., Nabavi S., Heidarali M. (2015). Intravenous magnesium sulfate: new method in prevention of contrast-induced nephropathy in primary percutaneous coronary intervention. Int. Urol. Nephrol..

[bib36] Fong J., Khan A. (2012). Hypocalcemia: updates in diagnosis and management for primary care. Canadian family physician Medecin de famille canadien.

[bib37] Ghidoli M., Colombo F., Sangiorgio S., Landoni M., Giupponi L., Nielsen E., Pilu R. (2021). Food containing bioactive flavonoids and other phenolic or sulfur phytochemicals with antiviral effect: can we design a promising diet against COVID-19?. Front. Nutr..

[bib39] Goff J.P. (2018). Invited review: mineral absorption mechanisms, mineral interactions that affect acid–base and antioxidant status, and diet considerations to improve mineral status. J. Dairy Sci..

[bib40] Gopinath B., Flood V.M., Wang J., Burlutsky G., Mitchell P. (2014). Lower dairy products and calcium intake is associated with adverse retinal vascular changes in older adults. Nutr. Metabol. Cardiovasc. Dis..

[bib41] Gopinath B., Flood V.M., Burlutsky G., Louie J.C.Y., Baur L., Mitchell P. (2014). Dairy food consumption, blood pressure and retinal microcirculation in adolescents. Nutr. Metabol. Cardiovasc. Dis..

[bib42] Grober U., Schmidt J., Kisters K. (2015). Magnesium in prevention and therapy. Nutrients.

[bib43] Guerrera M.P., Volpe S.L., Mao J.J. (2009). Therapeutic uses of magnesium. Am. Fam. Physician.

[bib44] Gülpınar Ö., Güçlü A.G. (2013). How to write a review article?. Turk J Urol.

[bib45] Gupta U.C., Gupta S.C. (2014). Sources and deficiency diseases of mineral nutrients in human health and nutrition: a review. Pedosphere.

[bib46] Hacker A.N., Fung E.B., King J.C. (2012). Role of calcium during pregnancy: maternal and fetal needs. Nutr. Rev..

[bib47] Hahm E.R., Singh S.V. (2014). Diallyl trisulfide inhibits estrogen receptor-alpha activity in human breast cancer cells. Breast Cancer Res. Treat..

[bib48] Hashemi G., Mirjalili M., Basiri Z., Tahamoli-Roudsari A., Kheiripour N., Shahdoust M., Ranjbar A., Mehrpooya M., Ataei S. (2019). A pilot study to evaluate the effects of oral N-acetyl cysteine on inflammatory and oxidative stress biomarkers in rheumatoid arthritis. Curr. Rheumatol. Rev..

[bib49] He J., Gu D., Chen J., Jaquish C.E., Rao D.C., Hixson J.E., Chen J.-c., Duan X., Huang J.-f., Chen C.-S., Kelly T.N., Bazzano L.A., Whelton P.K., GenSalt Collaborative Research G. (2009). Gender difference in blood pressure responses to dietary sodium intervention in the GenSalt study. J. Hypertens..

[bib50] Heaney R.P. (2006). Calcium intake and disease prevention. Arquivos Brasileiros Endocrinol. Metabol..

[bib51] Hemamy M., Pahlavani N., Amanollahi A., Islam S.M.S., McVicar J., Askari G., Malekahmadi M. (2021). Correction to: the effect of vitamin D and magnesium supplementation on the mental health status of attention-deficit hyperactive children: a randomized controlled trial. BMC Pediatr..

[bib52] Hibler E.A., Zhu X., Shrubsole M.J., Hou L., Dai Q. (2020). Physical activity, dietary calcium to magnesium intake and mortality in the National Health and Examination Survey 1999-2006 cohort. Int. J. Cancer.

[bib53] Hofmeyr G.J., Betran A.P., Singata-Madliki M., Cormick G., Munjanja S.P., Fawcus S., Mose S., Hall D., Ciganda A., Seuc A.H., Lawrie T.A., Bergel E., Roberts J.M., von Dadelszen P., Belizan J.M. (2019). Prepregnancy and early pregnancy calcium supplementation among women at high risk of pre-eclampsia: a multicentre, double-blind, randomised, placebo-controlled trial. Lancet.

[bib54] Holick M.F. (2006). Resurrection of vitamin D deficiency and rickets. J. Clin. Investig..

[bib55] Hunt C.D., Johnson L.K. (2006). Magnesium requirements: new estimations for men and women by cross-sectional statistical analyses of metabolic magnesium balance data. Am. J. Clin. Nutr..

[bib57] I. o. M. S. C. o. t. S. E. o. D. R. Intakes (1997).

[bib58] Jeong H., Jin H.S., Kim S.S., Shin D. (2020). Identifying interactions between dietary sodium, potassium, sodium-potassium ratios, andFGF5rs16998073 variants and their associated risk for hypertension in Korean adults. Nutrients.

[bib59] Jung S., Kim M.K., Shin J., Choi B.Y., Lee Y.H., Shin D.H., Shin M.H. (2019). High sodium intake and sodium to potassium ratio may be linked to subsequent increase in vascular damage in adults aged 40years and older: the Korean multi-rural communities cohort (MRCohort). Eur. J. Nutr..

[bib60] Kane R.E., Li A.P., Kaminski D.R. (1995). Sulfation and glucuronidation of acetaminophen by human hepatocytes cultured on Matrigel and type 1 collagen reproduces conjugation in vivo. Drug Metabol. Dispos.: the biological fate of chemicals.

[bib62] Kermani M.P., Nematy M. (2018). Maternal nutrition and the child's sex: a review. Int. J. Womens Health Reprod. Sci..

[bib63] Kiehn K.A., Mahoney J., Jones A.N., Hansen K.E. (2009). Vitamin D supplement intake in elderly fallers. J. Am. Geriatr. Soc..

[bib64] Kovacs C.S. (2018).

[bib65] Kovacs C., Ralston S. (2015). Presentation and management of osteoporosis presenting in association with pregnancy or lactation. Osteoporos. Int..

[bib66] Kruger M., Awan T., Poulsen R., Kuhn-Sherlock B. (2017).

[bib67] Krysiak R., Kowalcze K., Okopien B. (2019). Selenomethionine potentiates the impact of vitamin D on thyroid autoimmunity in euthyroid women with Hashimoto's thyroiditis and low vitamin D status. Pharmacol. Rep. : PRO.

[bib68] Lane-Cordova A.D., Scheider L.R., Tucker W., Cook J., Wilcox S., Liu J.H. (2019). Dietary sodium, potassium, and blood pressure in normotensive pregnant women: the national health and nutrition examination survey. Circulation.

[bib69] Lawson L.D., Hunsaker S.M. (2018). Allicin bioavailability and bioequivalence from garlic supplements and garlic foods. Nutrients.

[bib70] Leon J.B., Sullivan C.M., Sehgal A.R. (2013). The prevalence of phosphorus-containing food additives in top-selling foods in grocery stores. J. Ren. Nutr. : the official journal of the Council on Renal Nutrition of the National Kidney Foundation.

[bib71] Leonberg-Yoo A.K., Tighiouart H., Levey A.S., Beck G.J., Sarnak M.J. (2017). Urine potassium excretion, kidney failure, and mortality in CKD. Am. J. Kidney Dis. : the official journal of the National Kidney Foundation.

[bib72] Lete I., Mendoza N., de la Viuda E., Carmona F. (2018). Effectiveness of an antioxidant preparation with N-acetyl cysteine, alpha lipoic acid and bromelain in the treatment of endometriosis-associated pelvic pain: LEAP study. Eur. J. Obstet. Gynecol. Reprod. Biol..

[bib73] Li Y., Yin L., Peng Y.G., Liu X.Y., Cao X., Wang Y.Q., Yang P.T., Li X.H., Chen Z.H. (2020). The association of blood pressure with estimated urinary sodium, potassium excretion and their ratio in hypertensive, normotensive, and hypotensive Chinese adults, Asia Pac. J. Clin. Nutr..

[bib74] Liang Z., Chen L., McClafferty H., Lukowski R., MacGregor D., King J.T., Rizzi S., Sausbier M., McCobb D.P., Knaus H.G., Ruth P., Shipston M.J. (2011). Control of hypothalamic-pituitary-adrenal stress axis activity by the intermediate conductance calcium-activated potassium channel, SK4. J. Physiol..

[bib75] Lim H.S., Kim Y.J., Lee J., Yoon S.J., Lee B. (2020). Establishment of adequate nutrient intake criteria to achieve target weight loss in patients undergoing bariatric surgery. Nutrients.

[bib76] Liu H., Yu H., Xia J., Liu L., Liu G., Sang H., Peinemann F. (2020). Evidence-based topical treatments (azelaic acid, salicylic acid, nicotinamide, sulfur, zinc, and fruit acid) for acne: an abridged version of a Cochrane systematic review. J. Evidence-based Med..

[bib77] Lombardi G., Ziemann E., Banfi G., Corbetta S. (2020). Physical activity-dependent regulation of parathyroid hormone and calcium-phosphorous metabolism. Int. J. Mol. Sci..

[bib78] Maas A.H.E.M., Appelman Y.E.A. (2010). Gender differences in coronary heart disease. Neth. Heart J..

[bib79] Madeo B., Kara E., Cioni K., Vezzani S., Trenti T., Santi D., Simoni M., Rochira V. (2018). Serum calcium to phosphorous (Ca/P) ratio is a simple, inexpensive, and accurate tool in the diagnosis of primary hyperparathyroidism. JBMR plus.

[bib80] Marcus J.B., Marcus J.B. (2013). Culinary Nutrition.

[bib81] Medicine I.o. (2006).

[bib82] Merkiel S., Chalcarz W. (2016). Preschool diets in children from Pila, Poland, require urgent intervention as implied by high risk of nutrient inadequacies. J. Heatlh Popul. Nutr..

[bib83] Miekus N., Marszalek K., Podlacha M., Iqbal A., Puchalski C., Swiergiel A.H. (2020). Health benefits of plant-derived sulfur compounds. Glucosinolates, and Organosulfur Compounds, Molecules.

[bib84] Migdal K.U., Babcock M.C., Robinson A.T., Watso J.C., Wenner M.M., Stocker S.D., Farquhar W.B. (2020). The impact of high dietary sodium consumption on blood pressure variability in healthy, young adults. Am. J. Hypertens..

[bib85] Modem S., Dicarlo S.E., Reddy T.R. (2012). Fresh garlic extract induces growth arrest and morphological differentiation of MCF7 breast cancer cells. Genes & cancer.

[bib86] Mohan D., Yap K.H., Reidpath D., Soh Y.C., McGrattan A., Stephan B.C.M., Robinson L., Chaiyakunapruk N., Siervo M., De P.E.C.T. (2020). Link between dietary sodium intake, cognitive function, and dementia risk in middle-aged and older adults: a systematic review. J. Alzheimers Dis..

[bib87] Monti D.A., Zabrecky G., Kremens D., Liang T.-W., Wintering N.A., Cai J., Wei X., Bazzan A.J., Zhong L., Bowen B., Intenzo C.M., Iacovitti L., Newberg A.B. (2016). N-acetyl cysteine may support dopamine neurons in Parkinson's disease: preliminary clinical and cell line data. PLoS One.

[bib88] Morris A.L., Mohiuddin S.S. (2021).

[bib89] Moss H.G., Brown T.R., Wiest D.B., Jenkins D.D. (2018). N-Acetylcysteine rapidly replenishes central nervous system glutathione measured via magnetic resonance spectroscopy in human neonates with hypoxic-ischemic encephalopathy. J. Cerebr. Blood Flow Metabol. : official journal of the International Society of Cerebral Blood Flow and Metabolism.

[bib90] Musso C.G. (2009). Magnesium metabolism in health and disease. Int. Urol. Nephrol..

[bib91] Naz F., Raza A.B., Ijaz I., Kazi M.Y. (2014). Effectiveness of nebulized N-acetylcysteine solution in children with acute bronchiolitis. Journal of the College of Physicians and Surgeons--Pakistan : JCPSP.

[bib92] Newmark H.L., Heaney R.P., Lachance P.A. (2004). Should calcium and vitamin D be added to the current enrichment program for cereal-grain products?. Am. J. Clin. Nutr..

[bib93] Nielsen F.H. (2016). Guidance for the determination of status indicators and dietary requirements for magnesium. Magnes. Res..

[bib94] Nimni M.E., Han B., Cordoba F. (2007). Are we getting enough sulfur in our diet?. Nutr. Metabol..

[bib95] Oliai Araghi S., Kiefte-de Jong J.C., Trajanoska K., Koromani F., Rivadeneira F., Zillikens M.C., van Schoor N.M., de Groot L., Ikram M.A., Uitterlinden A.G., Stricker B.H., van der Velde N. (2020). Do vitamin D level and dietary calcium intake modify the association between loop diuretics and bone health?. Calcif. Tissue Int..

[bib96] Omotayo M.O., Dickin K.L., O'Brien K.O., Neufeld L.M., De Regil L.M., Stoltzfus R.J. (2016). Calcium supplementation to prevent preeclampsia: translating guidelines into practice in low-income countries. Advances in nutrition (Bethesda, Md).

[bib97] Onishi S., Yoshino S. (2006). Cathartic-induced fatal hypermagnesemia in the elderly. Intern. Med..

[bib98] Oria M., Harrison M., Stallings V.A. (2019).

[bib99] Ozdemir Z.C., Koc A., Aycicek A., Kocyigit A. (2014). N-Acetylcysteine supplementation reduces oxidative stress and DNA damage in children with beta-thalassemia. Hemoglobin.

[bib100] Pang K.S., Barker F., Simard A., Schwab A.J., Goresky C.A. (1995). Sulfation of acetaminophen by the perfused rat liver: the effect of red blood cell carriage. Hepatology.

[bib101] Park Y.M., Kwock C.K., Park S., Eicher-Miller H.A., Yang Y.J. (2018). An association of urinary sodium-potassium ratio with insulin resistance among Korean adults. Nutr. Res. Pract..

[bib102] Peacock M. (2010). Calcium metabolism in health and disease. Clin. J. Am. Soc. Nephrol. : CJASN.

[bib103] Pepe J., Colangelo L., Biamonte F., Sonato C., Danese V.C., Cecchetti V., Occhiuto M., Piazzolla V., De Martino V., Ferrone F., Minisola S., Cipriani C. (2020). Diagnosis and management of hypocalcemia. Endocrine.

[bib104] Perez V., Chang E.T. (2014). Sodium-to-potassium ratio and blood pressure, hypertension, and related factors. Advances in nutrition (Bethesda, Md).

[bib105] Pohl H.R., Wheeler J.S., Murray H.E. (2013). Sodium and potassium in health and disease. Metal ions in life sciences.

[bib106] Ravioli S., Gygli R., Funk G.C., Exadaktylos A., Lindner G. (2021). Prevalence and impact on outcome of sodium and potassium disorders in patients with community-acquired pneumonia: a retrospective analysis. Eur. J. Intern. Med..

[bib107] Reynolds R.M., Padfield P.L., Seckl J.R. (2006). Disorders of sodium balance. BMJ.

[bib108] Richardson H.W. (1997).

[bib109] Ross A.C., Manson J.E., Abrams S.A., Aloia J.F., Brannon P.M., Clinton S.K., Durazo-Arvizu R.A., Gallagher J.C., Gallo R.L., Jones G., Kovacs C.S., Mayne S.T., Rosen C.J., Shapses S.A. (2011). The 2011 dietary reference intakes for calcium and vitamin D: what dietetics practitioners need to know. J. Am. Diet Assoc..

[bib110] Ross A.C., Manson J.E., Abrams S.A., Aloia J.F., Brannon P.M., Clinton S.K., Durazo-Arvizu R.A., Gallagher J.C., Gallo R.L., Jones G. (2011). The 2011 report on dietary reference intakes for calcium and vitamin D from the Institute of Medicine: what clinicians need to know. J. Clin. Endocrinol. Metab..

[bib111] Sarris J., Byrne G.J., Bousman C., Stough C., Murphy J., MacDonald P., Adams L., Nazareth S., Oliver G., Cribb L., Savage K., Menon R., Chamoli S., Berk M., Ng C., Mischoulon D. (2018). Adjunctive S-adenosylmethionine (SAMe) in treating non-remittent major depressive disorder: an 8-week double-blind, randomized, controlled trial<sup/&gt. Eur. Neuropsychopharmacol : the journal of the European College of Neuropsychopharmacology.

[bib112] Sempos C.T., Durazo-Arvizu R.A., Fischer P.R., Munns C.F., Pettifor J.M., Thacher T.D. (2021). Serum 25-hydroxyvitamin D requirements to prevent nutritional rickets in Nigerian children on a low-calcium diet-a multivariable reanalysis. Am. J. Clin. Nutr..

[bib113] Shah C., Dai Q., Zhu X., Peek R.M., Smalley W., Roumie C., Shrubsole M.J. (2020). Associations between calcium and magnesium intake and the risk of incident gastric cancer: a prospective cohort analysis of the National Institutes of Health-American Association of Retired Persons (NIH-AARP) Diet and Health Study. Int. J. Cancer.

[bib114] Shah I.U., Sameen A., Manzoor M.F., Ahmed Z., Gao J., Farooq U., Siddiqi S.M., Siddique R., Habib A., Sun C., Siddeeg A. (2021). Association of dietary calcium, magnesium, and vitamin D with type 2 diabetes among US adults: National health and nutrition examination survey 2007-2014-A cross-sectional study. Food science & nutrition.

[bib115] Skinner J.D., Bounds W., Carruth B.R., Ziegler P. (2003). Longitudinal calcium intake is negatively related to children's body fat indexes. J. Am. Diet Assoc..

[bib116] Stamler J., Rose G., Stamler R., Elliott P., Dyer A., Marmot M. (1989). INTERSALT study findings. Public health and medical care implications. Hypertension.

[bib117] Stoll D.A., Muller A., Meinhardt A.K., Dotsch A., Greiner R., Kulling S.E., Huch M. (2020). Influence of salt concentration and iodized table salt on the microbiota of fermented cucumbers. Food Microbiol..

[bib119] Sun M., Wu X., Yu Y., Wang L., Xie D., Zhang Z., Chen L., Lu A., Zhang G., Li F. (2020). Disorders of calcium and phosphorus metabolism and the proteomics/metabolomics-based research. Front. Cell Dev. Biol..

[bib120] Teymoori F., Mokhtari E., Salehi P., Hosseini-Esfahani F., Mirmiran P., Azizi F. (2021). A nutrient pattern characterized by vitamin A, C, B6, potassium, and fructose is associated with reduced risk of insulin-related disorders: a prospective study among participants of Tehran lipid and glucose study. Diabetol. Metab. Syndrome.

[bib121] Thout S.R., Santos J.A., McKenzie B., Trieu K., Johnson C., McLean R., Arcand J., Campbell N.R.C., Webster J. (2019). The Science of Salt: updating the evidence on global estimates of salt intake. J. Clin. Hypertens..

[bib122] van der Waal S.V., Jiang L.M., de Soet J.J., van der Sluis L.W., Wesselink P.R., Crielaard W. (2012). Sodium chloride and potassium sorbate: a synergistic combination against Enterococcus faecalis biofilms: an in vitro study. Eur. J. Oral Sci..

[bib123] Verkaik-Kloosterman J., McCann M.T., Hoekstra J., Verhagen H. (2012).

[bib124] Vianello E., Dozio E., Barassi A., Sammarco G., Tacchini L., Marrocco-Trischitta M.M., Trimarchi S., Corsi Romanelli M.M. (2017). A pilot observational study on magnesium and calcium imbalance in elderly patients with acute aortic dissection. Immun. Ageing : Ir. Am..

[bib125] Vormann J. (2003). Magnesium: nutrition and metabolism. Mol. Aspect. Med..

[bib127] Wang D., Wang H., Han B., Wang B., Guo A., Zheng D., Liu C., Chang L., Peng M., Wang X. (2012). Sodium instead of potassium and chloride is an important macronutrient to improve leaf succulence and shoot development for halophyte Sesuvium portulacastrum. Plant Physiol. Biochem. : PPB (Plant Physiol. Biochem.).

[bib128] Wang H.C., Pao J., Lin S.Y., Sheen L.Y. (2012). Molecular mechanisms of garlic-derived allyl sulfides in the inhibition of skin cancer progression. Ann. N. Y. Acad. Sci..

[bib129] Weaver C.M. (2013). Potassium and health. Adv. Nutr..

[bib130] Wenzel U.O., Bode M., Kurts C., Ehmke H. (2019). Salt, inflammation, IL-17 and hypertension. Br. J. Pharmacol..

[bib131] Wiest D.B., Chang E., Fanning D., Garner S., Cox T., Jenkins D.D. (2014). Antenatal pharmacokinetics and placental transfer of N-acetylcysteine in chorioamnionitis for fetal neuroprotection. J. Pediatr..

[bib132] Winarno G.N.A., Pribadi A., Maruli H.J., Achmad E.D., Anwar R., Mose J.C., Nisa A.S., Trianasari N. (2021). Ratio of serum calcium to magnesium levels on pregnancy with and without preeclampsia. Med. Sci. Mon. Int. Med. J. Exp. Clin. Res. : international medical journal of experimental and clinical research.

[bib133] Wolak T., Shoham-Vardi I., Sergienko R., Sheiner E. (2016). High potassium level during pregnancy is associated with future cardiovascular morbidity. J. Matern. Fetal Neonatal Med..

[bib134] Woo H.W., Lim Y.-H., Kim M.K., Shin J., Lee Y.-H., Shin D.H., Shin M.-H., Choi B.Y. (2020). Prospective associations between total, animal, and vegetable calcium intake and metabolic syndrome in adults aged 40 years and older. Clin. Nutr..

[bib135] Yamaguchi Y., Kumagai H. (2020). Characteristics, biosynthesis, decomposition, metabolism and functions of the garlic odour precursor, S-allyl-L-cysteine sulfoxide. Exp. Ther. Med..

[bib136] Yee J., Rosenbaum D., Jacobs J.W., Sprague S.M. (2021). Small intestinal phosphate absorption: novel therapeutic implications. Am. J. Nephrol..

[bib137] Yilmaz Z.V., Akkas E., Turkmen G.G., Kara O., Yucel A., Uygur D. (2017). Dietary sodium and potassium intake were associated with hypertension, kidney damage and adverse perinatal outcome in pregnant women with preeclampsia. Hypertens. Pregnancy.

[bib138] Zhang L., Liu X.H., Qi H.B., Li Z., Fu X.D., Chen L., Shao Y. (2015). Ursodeoxycholic acid and S-adenosylmethionine in the treatment of intrahepatic cholestasis of pregnancy: a multi-centered randomized controlled trial. Eur. Rev. Med. Pharmacol. Sci..

[bib139] Zhang X., Li Y., Gobbo L.C.D., Rosanoff A., Wang J., Zhang W., Song Y. (2016). Effects of magnesium supplementation on blood pressure. Hypertension.

[bib140] Zhang Y., Lu L., Victor D.W., Xin Y., Xuan S. (2016). Ursodeoxycholic acid and S-adenosylmethionine for the treatment of intrahepatic cholestasis of pregnancy: a meta-analysis. Hepat. Mon..

